# Influence of Filler Material on the Microstructural and Mechanical Properties of 430 Ferritic Stainless Steel Weld Joints

**DOI:** 10.3390/ma16041590

**Published:** 2023-02-14

**Authors:** G. Shanmugasundar, Ankur Bansod, Vladimira Schindlerova, Robert Čep

**Affiliations:** 1 Department of Mechanical Engineering, Sri Sairam Institute of Technology, Chennai 600 044, India; 2 Department of Mechanical Engineering, Vel Tech Rangarajan Dr. Sagunthala R&D Institute of Science and Technology, Avadi 600 062, India; 3 Department of Mechanical Technology, Faculty of Mechanical Engineering, VSB-Technical University of Ostrava, 708 00 Ostrava, Czech Republic; 4 Department of Machining, Assembly and Engineering Metrology, Faculty of Mechanical Engineering, VSB-Technical University of Ostrava, 708 00 Ostrava, Czech Republic

**Keywords:** mechanical properties, filler material, microstructure, weld joints

## Abstract

Tungsten Inert Gas (TIG) welding is a commonly used welding technique for ferritic stainless steel, due to its ability to produce high-quality, clean, and precise welds. This welding method provides excellent control over the heat input, making it suitable for thin-walled, high-alloy materials such as ferritic stainless steel. The purpose of this study was to investigate the effect of using two different filler materials, 310 (austenitic) and 410 (ferritic), on the microstructural and mechanical properties of Tungsten Inert Gas (TIG) weld butt joints of 430 ferritic stainless steel (FSS). The results showed that the choice of filler material significantly impacted the dilution percentage, the chromium-nickel equivalent ratio, microstructure, microhardness, and tensile characteristics of the welded joint. The use of 310 filler resulted in a columnar microstructure, whereas the use of 410 filler resulted in a ferritic (acicular ferrite) microstructure with the presence of martensite and austenite. The sample welded with 410 filler demonstrated superior mechanical properties compared to the sample welded with 310 filler. These findings emphasize the importance of selecting the appropriate filler material in order to achieve the desired microstructural and mechanical properties in 430 FSS welded joints.

## 1. Introduction

Ferritic Stainless Steels (FSS) have ferrite-dominated microstructures and good corrosion resistances [1]. They are used in industries such as sugar, railway, paper, automobile, and chemical processing [2] and are classified into three categories based on their chromium content: low, medium, and high. Low chromium FSS, with a chromium content of 10.5% to 12.5%, are commonly used for manufacturing automotive components like exhaust tubes, where better corrosion resistance than carbon steel is needed. These grades are especially beneficial in the automotive industry as they provide a balance of both corrosion resistance and mechanical strength [3].

Tungsten Inert Gas (TIG) welding is an important process used to join FSS, and is known for producing welds with good mechanical properties [4,5,6,7]. The TIG welding process uses a non-consumable tungsten electrode to generate the weld, which is protected from oxidation and contamination by an inert gas shield. This results in a weld that is free of impurities and has a high-quality, clean appearance. TIG welding is also used in applications where a precise and clean weld is required, such as in the production of high-quality welds in the aerospace and automotive industries. The type of filler material used can greatly affect the mechanical and microstructural properties of the welds. Previous research has investigated the impact of various filler materials, including austenitic and ferritic stainless steel and Ni-based alloys, on the microstructural and mechanical properties of FSS weld joints. For instance, Mukherjee et al. found that using different filler wires (ER308L and ER316L) and heat inputs (0.4, 0.5, and 0.6 kJ/mm) in Gas Metal Arc Welding (GMAW) of SS409M resulted in varying levels of martensite formation and impact properties. Shojaati and Beidokhti [8] studied the effect of filler wires such as NiCr 80, austenitic, and duplex stainless steel on the mechanical properties and microstructures of dissimilar GMAW of SS304 and SS409. They discovered that austenitic stainless steel fillers resulted in a ferrite-austenite solidification mode, while Ni-based fillers decreased carbon segregation and reduced the diffusion rate of carbon [9]. The lower carbon diffusion rate in Ni-based filler metals results in improved weld quality and mechanical properties, making it a major advantage in welding applications where carbon presence can negatively impact the weld joint performance [10]. Lakshminarayanan et al. [11] conducted a study on the fatigue crack growth in welds made with different filler wires, including austenitic stainless steel (ASS), duplex stainless steel (DSS), and ferritic stainless steel (FSS). They found that the joint made with austenitic stainless steel had the highest impact toughness, followed by DSS and FSS.

Several studies have investigated the microstructure and mechanical properties of welded steel joints [12]. For example, researchers have analyzed the effect of process parameters on the hardness and microstructure of 304L-430 SS joints formed using laser beam welding [13]. Similar studies have been done on 409 stainless steel joints to evaluate the impact of different filler materials on microstructure and mechanical properties [14]. Kim et al. [15] studied the microstructure and mechanical properties of welded 316L stainless steel and found that the solidification path and microstructure of AISI 316L welds could be altered by altering the $Cr_{\mathrm{eq}}/Ni_{eq}$ ratio. The specimens had the best impact toughness at a $Cr_{\mathrm{eq}}/Ni_{eq}$ ratio of 1.3 under both as-weld and post-weld heat treatment conditions. Mukherjee and Pal [16] also found that the mechanical and microstructural behavior of welded stainless steel was significantly influenced by the $Cr_{\mathrm{eq}}/Ni_{eq}$ ratio and solidification mode. They noted that welding duplex stainless steel with GTAW does not result in chromium nitride formation in the weld metal, and that the weld core contains more austenite than the weld interface.

An important factor in selecting a filler material for welding FSS is its compatibility with the base material. Using mismatched filler materials can result in unfavorable microstructures and mechanical properties in the weld joint. For example, using an austenitic filler with a ferritic base material can lead to the formation of a brittle intermetallic compound called sigma phase, reducing the impact toughness of the weld joint [4]. On the other hand, using a ferritic filler with an austenitic base material can result in a two-phase microstructure, decreasing the ductility and tensile strength of the weld joint [8]. Hence, it is crucial to carefully consider the compatibility of the filler material with the base material to attain optimal microstructural and mechanical properties in the weld joint.

From the above discussion it clear that the choice of filler material in TIG welding has a significant impact on the microstructural and mechanical properties of Ferritic Stainless Steel weld joints. Previous research has shown that Ni-based filler metals have an advantage in welding applications, as they reduce the diffusion rate of carbon and improve weld quality and mechanical properties. The $Cr_{\mathrm{eq}}/Ni_{eq}$ ratio of the filler material, and the base material, play a crucial role in determining the microstructure and mechanical properties of the weld joint. The compatibility of the filler material with the base material is crucial, as using mismatched filler materials can result in unfavorable microstructures and mechanical properties. Hence, it is important to optimize the choice of filler material for specific applications by considering the impact of the filler material on the microstructural and mechanical properties of the weld joints, leading to improved performance and reliability.

This research aims to study the effect of filler material on the microstructural and mechanical properties of 430 ferritic stainless steel weld joints. By understanding this impact, the choice of filler material for specific applications can be optimized, resulting in improved performance and reliability.

The research paper is organized into distinct sections, each focusing on a specific aspect of the study. Section 1 provides background information on the topic, setting the stage for the rest of the paper. Section 2 provides details on the materials used and the welding process, including metallographic and microscopy, and mechanical testing. Section 3 presents the findings of the study, including dilution, the predictive model, microstructural characterization, XRD analysis, and mechanical testing results. The tensile and hardness tests are also discussed in detail. Finally, Section 4 summarizes the main findings and their implications.

## 2. Materials and Method

### 2.1. Materials and Welding

**Figure 1:**
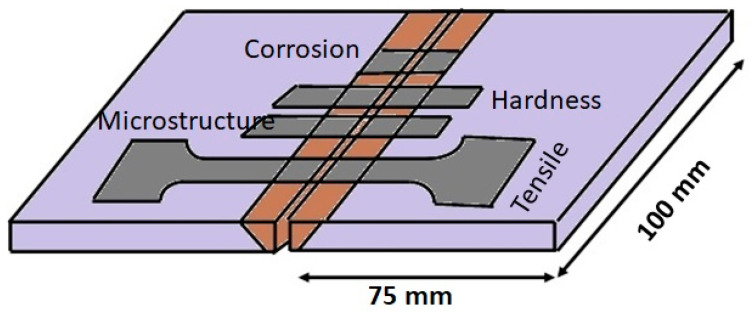
Schematic representation of welding specimens.

In this study, 430 FSS plates, in both as-rolled and mill-annealed conditions, were considered as the BM for welding. Plate samples of dimensions 100 × 75 × 3 mm in length × breadth × thickness, respectively, using an electrical discharge machine were created. TIG welding was performed using 2.14 mm-diameter 310 filler and 410 filler. The chemical compositions of the BM and filler materials are provided in Table 1. Square butt joints, with a plate gap of 1.2 mm, were completed using DCEN polarity and 110 A current. The heat input was computed using the current, arc voltage, and welding speed, as shown in Table 2. After cleaning the base plates with ethanol to remove any contaminants and prevent welding defects, manual welding was carried out. The pitting resistance equivalent number (PREN) was computed using Equation (1) [17]. Figure 1 illustrates the joint and sample schematic diagrams.

PREN = % Cr + 3.2% Mo + 16% N(1)

Similarly, the heat input was computed using Equation (2) [18].

(2) $$\mathrm{Heat}\;\mathrm{Input}\;\left(\mathrm{HI}\right)=\eta \frac{V\times I}{S\times 1000}\;\mathrm{KJ}/\mathrm{mm}$$

where I, V and S are the current (in amperes), voltage (in volts), and welding speed (in mm/s), respectively. The efficiency of TIG welding is assumed to be 0.6. There was a 7 lit/min gas flow. The heat input for both welding samples is displayed in Table 2. Weld sample cleanup was performed once welding was completed.

### 2.2. Metallographic and Microscopy

In accordance with ASTM E3-95, the metallographic samples were prepared by first polishing with 1200 grit emery paper, followed by cloth polishing with alumina slurry (0.75%). A mirror finish was achieved using 0.25% diamond paste [19]. The microstructures of the weld metal, heat-affected zone (HAZ), and base metal were examined using an optical microscope and a scanning electron microscope. The samples were etched using Marble’s reagent (4 g CuSO_4_ 5H_2_O, 20 mL HCl, 20 mL double-distilled water) for 10 s, and analyzed using a JEOL 6380A microscope. The ferrite content of the welds was measured using a Fischer Ferrito scope FMP30.

### 2.3. Mechanical Testing

The sample microhardness was determined using a Shimadzu Microhardness Tester (Kyoto, Japan), with a 500-g load applied for 10 s. Tensile testing was performed on the samples in accordance with ASTM E8M-04 by using a universal testing machine (Instron model 4467, Norwood, MA, USA). The mechanical properties of the weld joints were assessed based on these tests [20].

## 3. Results and Discussion

### 3.1. Dilution

**Figure 2:**
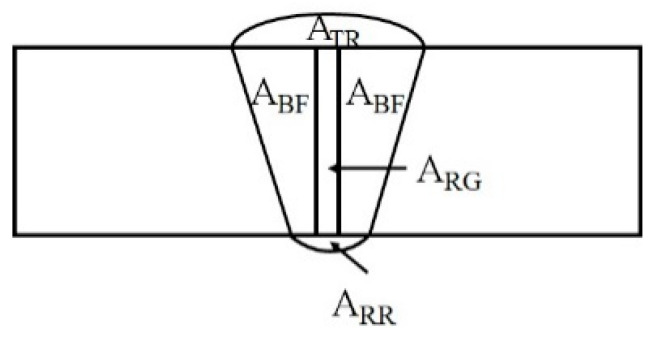
Schematic representation of a weld sample for calculation of dilution.

Figure 2 demonstrates a schematic representation for the calculation of the dilution in the weld joints. It was observed that the sample welded with 410 filler had a higher dilution compared to the sample welded with 310 filler. This may be the result of the low thermal expansion coefficient of ferritic filler materials. Additionally, it was observed that the chromium-to-nickel ratio increased with increasing percentage dilution. These results are in good agreement with those reported by Gupta et al. [21]. Table 3 provides the dilution values for both weld materials.

(3) $$A_{WD}=A_{TR}+A_{RR}+A_{RG}+A_{BF}$$

(4) $$DL\;\%=\left(A_{BF}/A_{WD}\right)\times 100$$

where $A_{TR}$ is the area of the top reinforcement, $A_{RR}$ is the area of the root reinforcement, $A_{RG}$ is the area of the root gap, and $A_{BF}$ is the area of the BM.

### 3.2. Predictive Model

A Schaeffler diagram was used as a predictive diagram to measure the amount of ferrite present in the weld metal. Also, it was used to predict the microstructure in the weldment. The $Cr_{\mathrm{eq}}$ and $Ni_{eq}$ were calculated according to Equations (5) and (6) [22].

(5) $$Cr_{\mathrm{eq}}=\%Cr+2\%Si+1.5\left(\%Mo\right)+5\left(\%V\right)+5.5\left(\%Al\right)+1.75\left(\%Nb\right)+1.5\left(\%Ti\right)+0.75\;\left(\%W\right)$$

(6) $$Ni_{eq}=\%Ni+\%Co+30\left(\%C\right)+25\left(\%N\right)+0.5\left(\%\;Mn\right)+0.3\left(\%Cu\right)\;$$

**Figure 3:**
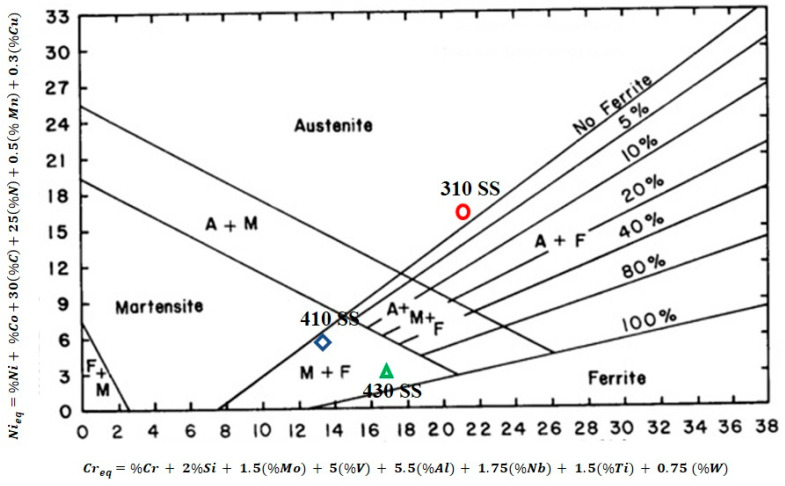
Schaeffler diagram.

**Figure 4:**
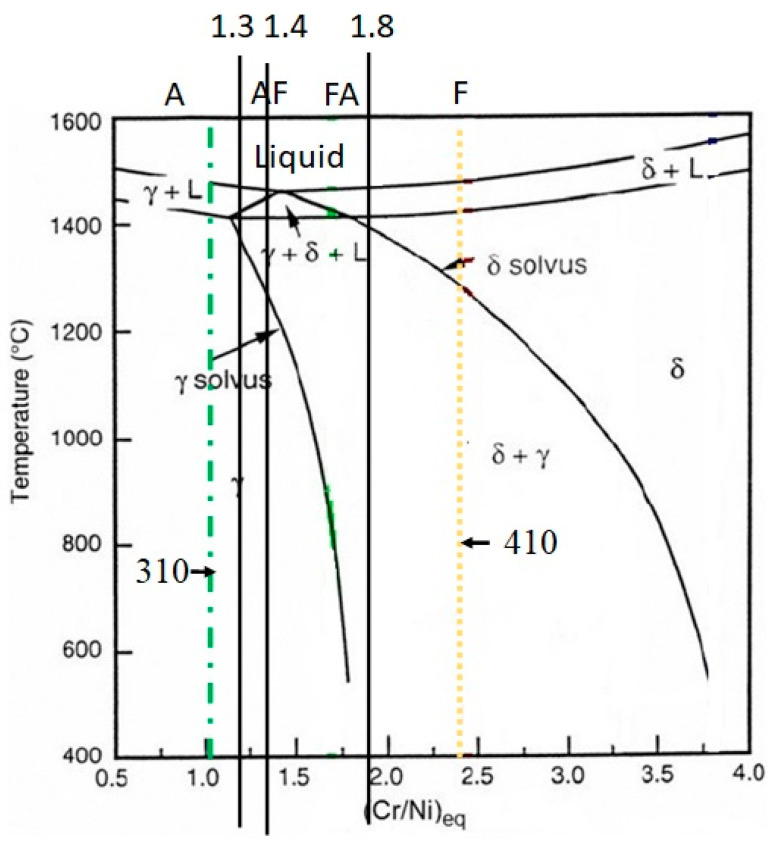
Fe-Ni-Cr Pseudo binary phase diagram.

Figure 3 displays the distinctive weld structure shape and various ferritic contents in the fusion zone. For the basic material, the $Cr_{\mathrm{eq}}$ and $Ni_{eq}$ were also calculated. Both of the filler materials have different microstructures, as seen in Figure 3. The microstructure of the connected weld sample (310 weld) mimics austenite. Additionally, it was discovered that the sample welded with 308L filler contained no ferrite, whereas the sample welded with 410 filler had a larger ferrite content compared to the 310 weld sample. The modes of solidification for both types of filler are shown in Figure 4. Due to a larger $Cr_{\mathrm{eq}}/Ni_{eq}$ value, the sample welded with the 310 electrode displays an austenitic structure. While austenite, acicular ferrite, and martensite were present in the sample welded with 410 filler. According to Equations (7)–(10), there are four solidification modes for following solid-state transformations.

The solidification mode for various modes is shown in Figure 4. The ‘A’ mode of solidification is indicated by a $Cr_{\mathrm{eq}}/Ni_{eq}$ value below 1.3, AF solidification is indicated by a value between 1.3 and 1.4, FA solidification is indicated by a value between 1.4 and 1.8, and F solidification is indicated by a value above 1.8. In the current study, it was discovered that the sample welded with 310 solidified in the A mode, but the sample welded with 410 filler solidified in the F mode. It is common knowledge that the presence of δ-ferrite lowers the possibility of solidification cracking in the weld zone. Additionally, while solidifying, δ-ferrite has the ability to absorb contaminants [6].

A-mode (Austenite) (*L* → *L* + *γ* → *γ*) (7)

AF-mode (Austenitic-ferritic) (*L* → *L* + *γ* → *L* + *δ* + *γ* → *γ* + *δ* (8)

(9) FA-mode (Ferritic-austenitic (FA) mode) (L→L+δ→L+δ+γ→γ+δ)

(10) F-mode (Ferritic) (L→L+δ→δ→δ+γ)

### 3.3. Microstructural Characterization

**Figure 5:**
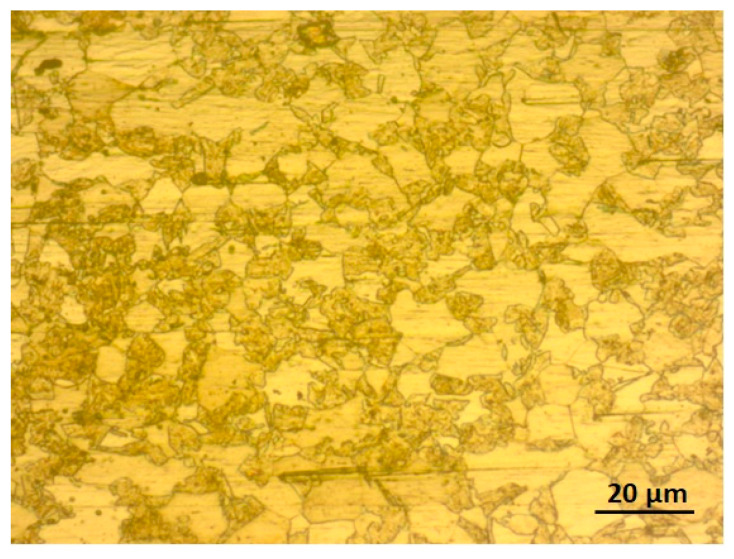
Optical micrograph of 430 SS.

**Figure 6:**
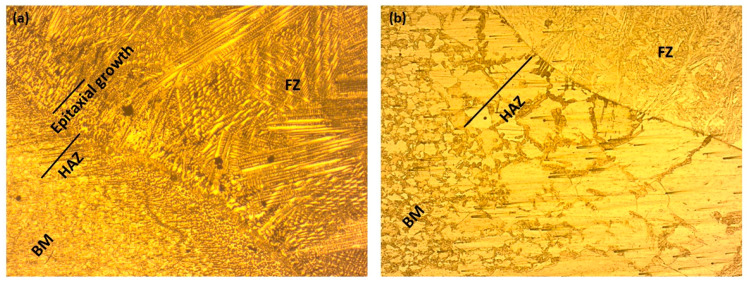
Microstructure of sample welded with (**a**) 310 (**b**) 410 filler.

**Figure 7:**
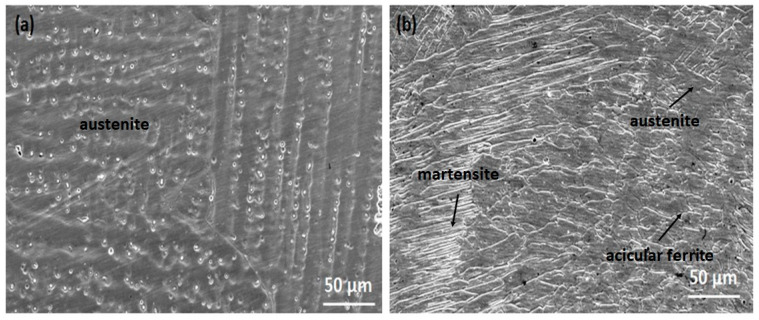
SEM Micrograph of weld zone of (**a**) 310 (**b**) 410 filler.

The microstructure of AISI 430 SS is shown in Figure 5. It can be seen that the BM microstructure contains some carbides and exhibits a fully ferritic structure. The average grain size of the base metal was found to be 10 µm and the grains are observed to be dispersed uniformly throughout the matrix. Figure 6a,b shows optical micrographs of both weld samples. Figure 7a,b shows the SEM micrographs of weld zone of both 310 and 410 filler. The two weld sample show different grain morphologies. Figure 6a shows the austenitic structure in the weld zone. As previously reported, the ($Cr_{\mathrm{eq}}/Ni_{eq}$) should be more than 1 for the primary δ solidification. However, in the present study, there is no primary δ solidification, therefore the solidification takes place from the γ austenite. The liquid transforms to γ + liquid on cooling. In this region, nucleation starts, and the liquid metal transforms to γ austenite. This nucleation further grows and forms the grains. Reducing the temperature transforms the remaining liquid metal to fully austenite. Due to the high Ni content in the filler, the transformation of the liquid metal to δ ferrite does not take place, as nickel is an austenitic stabilizer and hence promotes the formation of γ austenite in its microstructure. Also, the cooling rate affects the transformation in the weld structure. Next to the fusion zone, there is an epitaxial growth followed by the HAZ. It is observed that there is a chromium-depleted zone at the HAZ, followed by grain coarsening [18,23]. In the HAZ region, the grain size is bigger than the BM in the sample welded with 410 filler. An average grain size of 22 µm was observed for the HAZ, which is in fact larger than the size of BM. However, the sample welded with 308 filler shows a reasonable decrease in the grain size. This may be due to the change in the chemical composition of the phases, which may have caused the variation in the HAZ [24]. The average grain size of the HAZ welded with 310 filler was found to be 8 µm. It has been reported that the grain size in the microstructure has a huge impact on the mechanical properties. Fine grains in the microstructure result in higher hardness values and tensile strengths as compared to coarse grains [25]. In addition, thermal variation in the sample also controls the grain size to a large extent. It has been observed that samples kept for thermal aging for a longer duration result in a change in the microstructure to a coarse grain [26]. In the HAZ region, there may be some carbide precipitate in the vicinity of the grains. Figure 6b shows the microstructure of the 410 weld filler. The weld sample consists of austenite, martensite, and acicular ferrite in its microstructure. The electrode wire material (WM) used in this study solidified in the ferritic (F) phase because of its high chromium to nickel equivalent ratio ($Cr_{\mathrm{eq}}/Ni_{eq}$). In this mode, the molten liquid transforms to L+δ. The remaining liquid again transforms to the full δ structure. On further cooling, some of the δ ferrite transforms into γ austenite. Hence, the microstructure has δ+γ in the weld microstructure. In the ferrite grains of the AISI 430 stainless steel studied, a peppery structure was also observed, which may be indicative of precipitates within the microstructure of the HAZ. Also, no epitaxial growth was observed in the sample welded with 410 filler. Similar results were observed by Lippold [27]. The formation of a HAZ requires the presence of a temperature gradient [28]. It has been observed that ferritic steel tends to have more significant grain development in the HAZ compared to austenitic stainless steel. The presence of martensite in 430 BM can be predicted by using the Kaltenhauser (K factor) equivalency relationship, Equation (11) [29].

(11) K−factor =Cr+4Mo+6Si+2 A l+8Ti+40(C+N)−2 Mn−4Ni

When the K-factor, a measure of the cooling rate of a weld, exceeds 17.0, the likelihood of the formation of the martensite phase is low. In this study, the K-factor for AISI 430 was found to be greater than 13.4. Hence, there are chances for the formation of martensite in the weld made with 410 filler (ferritic filler). Table 4 shows the microstructural details of the welding. It was noted that the width of the HAZ in welds made with 410 filler was larger compared to those made with 310 filler.

### 3.4. XRD Analysis

**Figure 8:**
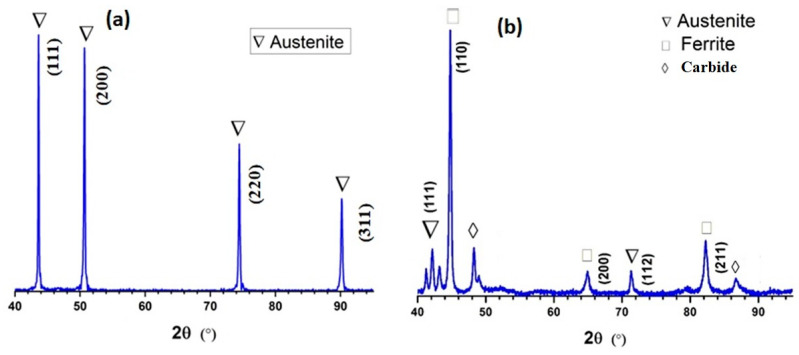
XRD analysis of weld sample (**a**) 310 SS (**b**) 410 SS.

Figure 8a,b shows the XRD patterns of 430 SS welded with 310 and 410 filler. The graphs depict several sharp peaks of different stages. The weld sample in Figure 8a has one set of peaks. Peaks indicative of austenite were observed when the sample was welded with 310 filler. As seen from the microstructure shown in Figure 6, the samples of 430 SS welded with 310 filler shows a columnar austenitic structure. The results of the XRD analysis correlate with the findings of the microstructural examination. Figure 8b shows the peaks of austenite, ferrite, and carbide. The formation of carbides in the weld structure is mainly due to the depletion of chromium at the grain boundaries of the HAZ, and the grains are found to be rich in chromium. This chromium depletion causes an increase in the width of the grain boundaries [30]. According to a study, a small amount of δ-ferrite is sufficient to prevent hot cracking during the solidification stage of the welding process. Fully austenitic stainless steel weld deposits are prone to micro fissuring as they cool [31].

### 3.5. Mechanical Testing

#### 3.5.1. Tensile Test

Tensile testing was conducted in the uniaxial direction (i.e., perpendicular to the welding direction) on three samples for each filler material. The average tensile test results are listed in Table 5. The average strength of the BM was found to be 515.2 MPa. It was observed that the sample welded with 410 filler had a higher tensile strength (732.4 MPa) compared to the sample welded with 310 filler (489 MPa). This difference in strength is likely due to the formation of ferrite grains in the weld structure, which improves the hardness of the weld sample; the hardness of the austenitic phase is generally lower as compared to the ferritic base metal. But the variation in strength would be mainly due to the dilution in the sample and variation in the $Cr_{\mathrm{eq}}/Ni_{eq}$ ratio. Similar results were observed by Verma et al. [32].

#### 3.5.2. Hardness Test

**Figure 9:**
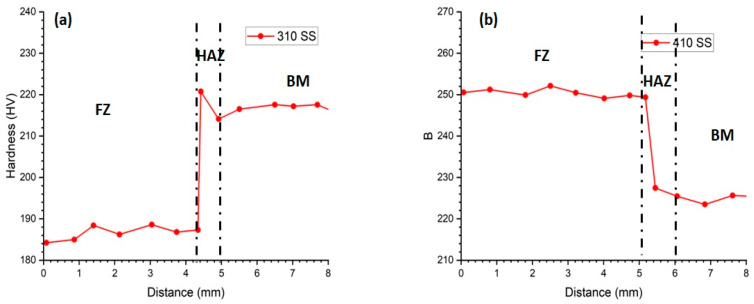
Microhardness profiles of (**a**) 310 SS filler and (**b**) 410 SS filler.

The microhardness profiles for both fillers (310 and 410), from the center of the weld metal to the BM, are shown in Figure 9a,b. The average hardness of the BM was found to be 221.1 HV0.5. The microhardness of the 310 filler welds exhibit a decreasing and then increasing trend with distance, while the 410 filler welds show a consistently decreasing trend. The average microhardness values in the fusion zone were 185.2 and 250.6 HV0.5 for fillers 310 and 410, respectively. This difference in microhardness can be attributed to the different solidification modes (A and F) and the differences in electrode composition, as the 410 filler has a higher $Cr_{\mathrm{eq}}/Ni_{eq}$ ratio, leading to a higher ferrite content and higher microhardness compared to the fully austenitic structure of the 310 filler. In the heat-affected zone, there is a sharp increase in the hardness value, as shown in Figure 9a. This may be due to the epitaxial growth in the 310 filler weld, which leads to a sudden increase in the hardness.

## 4. Conclusions

The following conclusions can be drawn from the above investigations—

- Both 410 and 310 filler materials can be successfully used for TIG welding of 430 ferritic stainless steel.
- The microstructure of the welds made using 410 filler were found to have ferrite, martensite, and austenite, and thus show a higher $Cr_{\mathrm{eq}}/Ni_{eq}$ ratio. On the other hand, the welds made using 310 filler were found to have a reduced $Cr_{\mathrm{eq}}/Ni_{eq}$ ratio, and an austenitic microstructure.
- Epitaxial growth was observed in the sample welded with 310 filler. However, no such growth was observed in the sample welded with the ferritic filler (410).
- Different behaviors are displayed by different alloy solidification modes and electrode compositions. These significantly affect the weld specimen’s hardness and tensile strength. Higher $Cr_{\mathrm{eq}}/Ni_{eq}$ ratios result in the ferritic mode, where the austenite-dominated (310 filler) phase was significantly outperformed by the ferrite-dominated (410 filler) phase in terms of mechanical properties.
- The tensile strength was found to be higher in samples welded with 410 filler as compared to those welded with 310 filler.
- The hardness of the 410 weld samples was higher than the 310 weld samples. This is due to the formation of δ ferrite in the weld zone.

The limitations of this study include a limited examination of the filler materials and their impact on the properties of TIG welded 430 ferritic stainless steel. Furthermore, the mechanical tests performed were limited to tensile testing and hardness testing, and other forms of testing such as fatigue testing, corrosion testing, and impact testing were not conducted. In terms of the predictive model, further research could be done to improve its accuracy and to examine its reliability in predicting the properties of TIG welded joints.

In terms of future scope, the findings of this research could be extended to examine the effect of different welding parameters, such as welding speed and current, on the microstructural and mechanical properties of the welds. In addition, other filler materials could be examined to determine their impact on the properties of TIG welded 430 ferritic stainless steel. Additionally, the findings of this research could be applied to other types of stainless steels to determine the effect of the filler material selection on the properties of TIG welded joints of those materials.

## Figures and Tables

**Table 1:** Chemical composition of various materials.

Materials	Composition (wt. %)										
	C	Si	Mn	Cr	Mo	Ni	Fe	$Cr_{\mathrm{eq}}$	$Ni_{eq}$	$Cr_{\mathrm{eq}}/Ni_{eq}$	PREN
430 (BM)	0.08	0.34	0.58	16.28	0	0.13	Balance	16.96	2.82	6.01	16.28
410 Filler	0.06	0.71	0.82	17.01	0.05	5.23	Balance	18.49	7.44	2.48	17.16
310 Filler	0.1	0.49	1.5	26.10	0	21.5	Balance	26.98	25.25	1.06	26.01

**Table 2:** Welding process parameters for both the samples.

S. No.	Sample Specification	Current (I)	Voltage (V)	Welding Speed (S)	Heat Input (HI)	Filler Diameter (mm)
1	BM (430) welded with 310 filler	110	20	3.8	0.347	2.14
2	BM (430) welded with 410 filler	110	20	3.4	0.388	2.14

**Table 3:** Dilution for different fillers in welding.

S. No.	Sample Specification	*A_BF_*/mm^2^	*A_WD_*/mm^2^	DL % = (*A_BF_*/*A_WD_*) × 100
1	BM (430) welded with 310 filler	12.26	36.20	33.86
2	BM (430) welded with 410 filler	15.10	35.10	43.01

**Table 4:** Microstructural details of welding.

Sample	Unmixed Zone Length (µm)	HAZ Length (µm)	δ-Ferrite by Ferritoscope (FN)
310 weld zone	86	95	0.10
410 weld zone	-	213	6.4

**Table 5:** Mechanical properties of various samples.

Sample	Ultimate Tensile Strength (MPa)	Standard Deviation (MPa)	% Elongation	Failure
430 BM	515.2	13	30	-
430 SS with 310 filler	489.5	75	32	Weld joint
430 SS with 410 filler	732.4	45	22	Weld joint

## Data Availability

The data presented in this study are available in the article.

## References

[1] Bansod A.V., Khobragade N.N., Giradkar K.V., Patil A.P. (2017). Effect of concentration of hyaluronic acid and NaCl on corrosion behavior of 316L austenitic stainless steel. Mater. Res. Express.

[2] Mamphekgo T.C., Matjeke V.J., Pillay K. (2018). Investigation of abnormal corrosion of 10.5–12.5 chromium ferritic stainless steel used to fabricate railway coal wagons. IOP Conf. Ser. Mater. Sci. Eng..

[3] Li C.-X., Dang S.-H., Wang L.-P., Zhang C.-L., Han P.-D. (2014). Effect of Cr, Mo, and Nb additions on intergranular cohesion of ferritic stainless steel: First-principles determination. Chin. Phys. B.

[4] Meng X., Qin G., Zhang Y., Fu B., Zou Z. (2014). High speed TIG–MAG hybrid arc welding of mild steel plate. J. Mater. Process. Technol..

[5] Venugopal A., Sreekumar K., Raja V.S. (2012). Stress Corrosion Cracking Behavior of Multipass TIG-Welded AA2219 Aluminum Alloy in 3.5 wt pct NaCl Solution. Met. Mater. Trans. A.

[6] Yan J., Gao M., Zeng X. (2009). Study on microstructure and mechanical properties of 304 stainless steel joints by TIG, laser and laser-TIG hybrid welding. Opt. Lasers Eng..

[7] Serindağ H.T., Çam G. (2022). Multi-pass butt welding of thick AISI 316L plates by gas tungsten arc welding: Microstructural and mechanical characterization. Int. J. Press. Vessel. Pip..

[8] Shojaati M., Beidokhti B. (2017). Characterization of AISI 304/AISI 409 stainless steel joints using different filler materials. Constr. Build. Mater..

[9] Parker J., Stratford G. (2001). The high-temperature performance of nickel-based transition joints: I. Deformation behaviour. Mater. Sci. Eng. A.

[10] Samal M., Seidenfuss M., Roos E., Balani K. (2011). Investigation of failure behavior of ferritic–austenitic type of dissimilar steel welded joints. Eng. Fail. Anal..

[11] Lakshminarayanan A., Shanmugam K., Balasubramanian V. (2009). Fatigue Crack Growth Behavior of Gas Metal Arc Welded AISI 409 Grade Ferritic Stainless Steel Joints. J. Mater. Eng. Perform..

[12] Hsieh C.-C., Lin D.-Y., Chen M.-C., Wu W. (2008). Precipitation and strengthening behavior of massive δ-ferrite in dissimilar stainless steels during massive phase transformation. Mater. Sci. Eng. A.

[13] Khan M., Romoli L., Fiaschi M., Dini G., Sarri F. (2012). Laser beam welding of dissimilar stainless steels in a fillet joint configuration. J. Mater. Process. Technol..

[14] Shanmugam K., Lakshminarayanan A., Balasubramanian V. (2009). Effect of weld metal properties on fatigue crack growth behaviour of gas tungsten arc welded AISI 409M grade ferritic stainless steel joints. Int. J. Press. Vessel. Pip..

[15] Kim Y., Lee D., Byun J., Jung K., Kim J., Lee H., Shin Y., Kim S. (2011). The effect of sigma phases formation depending on Cr/Ni equivalent ratio in AISI 316L weldments. Mater. Des..

[16] Mukherjee M., Pal T. (2012). Influence of Heat Input on Martensite Formation and Impact Property of Ferritic-Austenitic Dissimilar Weld Metals. J. Mater. Sci. Technol..

[17] Kang D., Lee H. (2013). Study of the correlation between pitting corrosion and the component ratio of the dual phase in duplex stainless steel welds. Corros. Sci..

[18] Bansod A.V., Patil A.P., Verma J., Shukla S. (2019). Microstructure, Mechanical and Electrochemical Evaluation of Dissimilar low Ni SS and 304 SS using Different Filler Materials. Mater. Res..

[19] Standard Practice for Preparation of Metallographic Specimens. https://www.astm.org/e0003-95.html.

[20] (2010). Tension Testing of Metallic Materials.

[21] Gupta S.K., Raja A.R., Vashista M., Yusufzai M.Z.K. (2018). Effect of heat input on microstructure and mechanical properties in gas metal arc welding of ferritic stainless steel. Mater. Res. Express.

[22] Verma J., Taiwade R.V., Khatirkar R.K., Sapate S.G., Gaikwad A.D. (2016). Microstructure, Mechanical and Intergranular Corrosion Behavior of Dissimilar DSS 2205 and ASS 316L Shielded Metal Arc Welds. Trans. Indian Inst. Met..

[23] Bansod A.V., Patil A.P., Moon A.P., Khobragade N.N. (2016). Intergranular Corrosion Behavior of Low-Nickel and 304 Austenitic Stainless Steels. J. Mater. Eng. Perform..

[24] Oh D., Han K., Hong S., Lee C. (2011). Effects of alloying elements on the thermal fatigue properties of the ferritic stainless steel weld HAZ. Procedia Eng..

[25] Opiela M., Fojt-Dymara G., Grajcar A., Borek W. (2020). Effect of Grain Size on the Microstructure and Strain Hardening Behavior of Solution Heat-Treated Low-C High-Mn Steel. Materials.

[26] Moshtaghi M., Loder B., Safyari M., Willidal T., Hojo T., Mori G. (2022). Hydrogen trapping and desorption affected by ferrite grain boundary types in shielded metal and flux-cored arc weldments with Ni addition. Int. J. Hydrogen Energy.

[27] Lippold J.C., Kotecki D.J. (2005). Welding Metallurgy and Weldability of Stainless Steels.

[28] Mukherjee M., Dutta A., Kanjilal P., Pal T.K., Sisodia S. (2015). Enhancement of Microstructural and Mechanical Properties by Pulse Mode of Metal Transfer in Welded Modified Ferritic Stainless Steel. ISIJ Int..

[29] Alizadeh-Sh M., Marashi S., Pouranvari M. (2014). Resistance spot welding of AISI 430 ferritic stainless steel: Phase transformations and mechanical properties. Mater. Des..

[30] Yin Y., Faulkner R.G., Moreton P., Armson I., Coyle P. (2010). Grain boundary chromium depletion in austenitic alloys. J. Mater. Sci..

[31] Dadfar M., Fathi M., Karimzadeh F., Saatchi A. (2007). Effect of TIG welding on corrosion behavior of 316L stainless steel. Mater. Lett..

[32] Verma J., Taiwade R.V. (2016). Effect of Austenitic and Austeno-Ferritic Electrodes on 2205 Duplex and 316L Austenitic Stainless Steel Dissimilar Welds. J. Mater. Eng. Perform..

